# Convergence of Cannabis and Psychosis on the Dopamine System

**DOI:** 10.1001/jamapsychiatry.2025.0432

**Published:** 2025-04-09

**Authors:** Jessica Ahrens, Sabrina D. Ford, Betsy Schaefer, David Reese, Ali R. Khan, Philip Tibbo, Rachel Rabin, Clifford M. Cassidy, Lena Palaniyappan

**Affiliations:** 1 Integrated Program in Neuroscience, McGill University, Montreal, Quebec, Canada; 2 Douglas Mental Health University Institute, Department of Psychiatry, McGill University, Montreal, Quebec, Canada; 3 Robarts Research Institute, London, Ontario, Canada; 4 Lawson Health Research Institute, London, Ontario, Canada; 5 Department of Medical Biophysics, Schulich School of Medicine and Dentistry, Western University, London, Ontario, Canada; 6 Department of Psychiatry, Dalhousie University, Halifax, Nova Scotia, Canada; 7 Renaissance School of Medicine, Stony Brook University, Stony Brook, New York

## Abstract

**Importance:**

Despite evidence that individuals with a cannabis use disorder (CUD) are at elevated risk of psychosis and that the neurotransmitter dopamine has a role in psychosis, the mechanism linking cannabis use and psychosis remains unclear.

**Objective:**

To use neuromelanin-sensitive magnetic resonance imaging (MRI), referred to as the *neuromelanin-MRI signal*, a practical, proxy measure of dopamine function, to assess whether a common alteration in the dopamine system may be implicated in CUD and psychosis and whether this alteration can be observed in those with a CUD whether or not they have a diagnosis of first-episode schizophrenia (FES).

**Design, Setting, and Participants:**

This longitudinal observational cohort study recruited individuals from 2019 to 2023 from an early psychosis service and the surrounding communities in London, Ontario. The sample included individuals with and without CUD, with some in each group also diagnosed with FES.

**Exposures:**

FES and CUD diagnoses from the Structured Clinical Interview for *DSM-5*.

**Main Outcomes and Measures:**

Neuromelanin-MRI signals within the midbrain (substantia nigra [SN]/ventral tegmental area [VTA]) including a subregion previously linked to the severity of untreated psychosis (a priori region of interest). Linear mixed-effects analyses were performed relating neuromelanin-MRI signals to clinical measures.

**Results:**

A total of 36 individuals without CUD (mean [SD] age, 22.3 [3.2] years; 29 male [81%]; 12 with FES) and 25 individuals with CUD (mean [SD] age, 24.3 [4.7] years; 22 male [88%]; 16 with FES) participated in the study. One-year follow-up was completed for 12 individuals with CUD and 25 without CUD. CUD was associated with elevated neuromelanin-MRI signal in a set of ventral SN/VTA voxels (387 of 2060 SN/VTA voxels, corrected *P* = .03, permutation test). CUD was also associated with elevated neuromelanin-MRI signal in the psychosis-related region of interest (*t*_92_ = 2.12, *P* = .04) with a significant dose-dependent association (higher burden of CUD symptoms associated with higher neuromelanin-MRI signal, *F*_1, 96_ = 4.89; *P* = .03). In contrast, participants with FES did not exhibit a significant elevation in neuromelanin-MRI signal (241 SN/VTA voxels had elevated signal, corrected *P* = .09). There was no association between time and neuromelanin-MRI signal.

**Conclusions and Relevance:**

Elevated dopamine function in a critical SN/VTA subregion may be associated with psychosis risk in people with CUD. Cannabis was associated with the hypothesized final common pathway for the clinical expression of psychotic symptoms.

## Key Points

**Question:**

Is cannabis associated with the same midbrain dopamine pathway involved in psychosis?

**Findings:**

In this cohort study including 61 individuals, participants with cannabis use disorder exhibited increased neuromelanin–magnetic resonance imaging signals in specific voxels of the substantia nigra/ventral tegmental area (SN/VTA). This subregion has previously shown elevated signals associated with untreated psychotic symptoms.

**Meaning:**

Increased dopamine functioning in the SN/VTA may be associated with the risk of psychosis in people with cannabis use disorder.

## Teaser

This cohort study investigates the association between cannabis and the midbrain dopamine pathway involved in psychosis.

## Introduction

Cannabis use disorder (CUD) is a common problem that is of particular concern due to the association between cannabis and psychosis.^1,2^ In healthy individuals, administration of Δ9-tetrahydrocannabinol (THC), the main psychoactive compound in cannabis, induces positive psychotic symptoms, such as suspiciousness, delusions, and altered perception^3,4,5^ and negative symptoms, including blunted affect and amotivation.^6^ Higher levels of cannabis use are consistently associated with an increased risk of psychosis; this dose-response relationship supports a causal influence.^6^ Individuals with psychosis who use cannabis report an earlier illness onset compared with those who do not.^7^ However, the mechanism through which cannabis may contribute to psychotic symptoms remains unclear and much debated,^8^ especially as cannabis use is neither sufficient nor necessary to cause persistent psychotic disorders.^9^ One possibility is that cannabis affects the same final common pathway of dopaminergic excess in psychosis.^10,11^ Importantly, given its association with psychotic relapses in patients with schizophrenia,^12^ its effect on dopamine may be dose dependent and sustained when use is continued.

The dopamine hypothesis of schizophrenia states that striatal hyperdopaminergia underlies the positive psychotic symptoms of schizophrenia.^13^ The effect of cannabis use on the dopaminergic system is less well known. Following THC administration, dopamine is released in striatal and cortical brain areas,^14,15^ an effect similar to dopamine levels reported in psychosis and schizophrenia.^16^ In contrast, positron emission tomography (PET) studies in people with cannabis use have found reduced dopamine synthesis capacity^17^ and release in the striatum.^18^ Therefore, no consistent dopamine availability changes have been demonstrated in cannabis users to date. The reported differences in short-term vs persistent effects of cannabis use on dopamine further complicate the mechanistic link connecting cannabis, dopamine, and schizophrenia.

Neuromelanin-sensitive magnetic resonance imaging (referred to as *neuromelanin-MRI*) is a novel approach with noninvasive, short acquisition periods that provides an indirect index of dopamine from the substantia nigra (SN, including the ventral tegmental area [VTA]; SN/VTA) where most dopaminergic cells originate.^19^ Neuromelanin, a breakdown product of cytosolic dopamine, accumulates in these neurons by forming insoluble complexes with iron.^20^ The resulting paramagnetic properties create an endogenous localized contrast in the MRI, quantified as contrast-to-noise ratio (CNR), in comparison with a nondopaminergic reference region.^21^ Neuromelanin-MRI signal is widely regarded as a trait marker reflecting long-term processes.^22^ Across the life span, neuromelanin accumulates gradually, leading to an age-related increase of the neuromelanin-MRI signal, before dropping with the onset of neurodegeneration.^20,23,24^ Disruptive factors such as oxidative stress or toxic insults may influence neuromelanin levels, but these changes occur over long timescales, marking cumulative neuronal history.^25^

Higher neuromelanin-MRI signals occur in disorders where hyperdopaminergia is suspected, such as schizophrenia^19^ and cocaine use disorder.^26^ In schizophrenia, a subset of voxels in the SN/VTA show higher neuromelanin-MRI signal that correlates with psychosis severity.^19^ Additionally, recent research indicates that SN/VTA neuromelanin-MRI signal is associated with elevated dopamine synthesis capacity of the striatum^27^ and striatal dopamine release capacity.^19^ Taken together, SN/VTA neuromelanin-MRI signals may reflect neuromelanin accumulation that is driven by dopamine synthesis in the neurons projecting to the striatum. Thus, the neuromelanin-MRI signal of the SN/VTA provides a viable method for examining the putative dopaminergic effects of cannabis use in relation to schizophrenia. To our knowledge, the effect of CUD on neuromelanin-MRI signals in the SN/VTA or its subregions with psychosis-related excess in dopaminergic turnover has not yet been studied.

We investigated if the neuromelanin-MRI signal is higher in those with CUD compared with those without CUD, and if CUD and first-episode schizophrenia (FES) have an additive outcome of higher neuromelanin-MRI signal. We also examined the longitudinal association of CUD with neuromelanin over 1 year, with the primary aim of determining the extent to which persisting cannabis use is associated with the SN/VTA neuromelanin-MRI signal. We hypothesize that (1) the SN/VTA neuromelanin-MRI signal will be higher in individuals with CUD compared with individuals without CUD, especially in regions previously shown to be related to psychosis severity; (2) neuromelanin-MRI signal abnormalities in FES and CUD will interact with an additive outcome (ie, higher CUD-associated neuromelanin in the group with FES than in the group without FES); and (3) the neuromelanin-MRI signal will remain elevated over 1 year in individuals with CUD compared with individuals without CUD, indicating a persistent predisposition to psychosis.

## Methods

### Participants

This longitudinal observational cohort study, Cannabis Effects on White Matter, Microstructure, and Outcomes in Early Phase Psychosis, was approved by Western University’s health science research ethics board. All study participants signed written informed consent. Participants aged 18 to 35 years were recruited from London, Ontario, between 2019 and 2023. The sample included patients with FES (with and without CUD) who were within 6 months of entry to a specialized Early Intervention Program for Psychosis. Additionally, sex- and age-matched nonclinical community volunteers (with and without CUD) from the same locality were recruited. Race and ethnicity data were not gathered for this study as these variables were not included in the original protocol. The Structured Clinical Interview for *DSM* Disorders was administered by a research psychiatrist (L.P.) to diagnose schizophrenia and CUD and to rule out current alcohol and stimulant drug use disorder. Nonclinical volunteers with CUD were excluded if they had a first-degree family history of schizophrenia or bipolar illness. Notably, the participants without CUD were not cannabis naive; thus, they were representative of the general population that shared many characteristics with the group of participants with CUD, except for the CUD status. This study followed the Strengthening the Reporting of Observational Studies in Epidemiology (STROBE) reporting guidelines.

### Clinical and Cognitive Measures

Psychotic symptoms were assessed using the Positive and Negative Syndrome Scale (PANSS^28^) (Table) for all participants, followed by cognitive assessments with National Adult Reading Test (NART^29^), the written digit symbol substitution test (W-DSST) and oral digit symbol substitution test (O-DSST), the mean of which is reported together as modified DSST,^30^ and the Category Fluency Test (Table) at baseline, 6 months, and 12-month follow-up. THC levels were quantified on the day of scanning with the passive drool method (Quantisal device [Immunalysis]) using commercially available liquid chromatography-mass spectrometry (East Coast Mobile Medical Inc). Details of questionnaire-based substance use assessments can be found in the eMethods in Supplement 1.

**Table.** Demographics and Clinical Characteristics of Participants

Characteristic	No. (%)		*P *value
	No CUD (n = 36)	CUD (n = 25)	
Sex			
Female	7 (19.44)	3 (12.00)	.44
Male	29 (80.60)	22 (88.00)	
First-episode schizophrenia	12 (33.30)	16 (64.00)	.06
Alcohol frequency			
Less than weekly	27 (75.00)	21 (84.00)	.92
Greater than weekly	9 (25.00)	2 (8.00)	
CUD severity^a^			
None	29 (100)	0	<.001
Mild	0	6 (24.00)	
Moderate	0	6 (24.00)	
Severe	0	11 (48.00)	
Past-month cannabis use^a^			
No use	21 (58.33)	1 (4.00)	<.001
Less than monthly	0	0	
1-2 Times per month	9 (25.00)	0	
Less than weekly	1 (2.78)	0	
1-2 Times per week	1 (2.78)	0	
Every other day	3 (8.33)	1 (4.00)	
Daily	1 (2.78)	21 (84.00)	
Cannabis use on your own			
Never	27 (75.00)	0	<.001
Rarely	1 (2.78)	3 (12.00)	
Time to time	1 (2.78)	2 (8.00)	
Quite often	3 (8.33)	8 (32.00)	
Very often	4 (11.11)	12 (48.00)	
Problems because of cannabis use			
Never	35 (97.22)	19 (76.00)	.08
Rarely	0	4 (16.00)	
Time to time	0	0	
Quite often	1 (2.78)	2 (8.00)	
Very often	0	0	
Age, mean (SD), y	22.33 (3.21)	24.30 (4.71)	.06
Education, mean (SD), y	14.83 (2.58)	13.89 (2.77)	.18
Premorbid IQ, mean (SD)	113.30 (5.15)	109.42 (5.57)	.007
Category fluency, mean (SD)^b^	25.83 (5.86)	22.00 (5.86)	.01
DSST score, mean (SD)	64.47 (12.23)	6.91 (9.29)	.22
Nicotine use, mean (SD), cigarettes/d	0.51 (2.87)	3.30 (6.01)	.005
Salivary THC levels, mean (SD), ng/mL^c^	2.53 (10.20)	17.47 (33.70)	.03
Age of regular cannabis use onset, mean (SD),^d^ y	17.27 (3.51)	17.64 (2.93)	.63
SUQ cannabis total score from 0-24, mean (SD)	1.73 (2.81)	1.56 (3.22)	<.001
SUQ total score from 0-50, mean (SD)	6.65 (4.64)	17.40 (5.43)	<.001

Abbreviations: CUD, cannabis use disorder; DSST, digit symbol substitution test; THC, tetrahydrocannabinol; SUQ, substance use questionnaire.

^a^ Two participants with CUD had no information pertaining to level of severity or use in the past month.

^b^ Category fluency score is the total number of correct words in 60 seconds.

^c^ Participants without CUD n = 30, of which 3 had nonzero THC values and participants with CUD n = 15, of which 8 had nonzero THC values.

^d^ Participants without CUD n = 22; participants with CUD n = 23.

### MRI Acquisition

MRI scans were acquired for all participants on a 3-T GE Discovery MR750 using a 32-channel, phased-array head coil at baseline and 1-year follow-up. Neuromelanin-MRI images were collected via a 2-dimensional gradient echo sequence with the following parameters: repetition time (TR) = 285 milliseconds, echo time (TE) = 3.9 milliseconds, flip angle = 40°, field of view (FOV) = 220 × 165 mm^2^, number of slices = 10, slice thickness = 3.0 mm, number of averages = 8, acquisition time = 12.16 minutes, and in-plane resolution = 0.43 × 0.43 mm. The image stack was oriented along the anterior commissure–posterior commissure line, providing coverage of the SN/VTA-containing portions of the midbrain (and structures surrounding the brainstem). Whole-brain, high-resolution structural MRI scans were acquired for preprocessing of the neuromelanin-MRI data: a T1-weighted 3-dimensional spoiled gradient recalled echo sequence (inversion time = 400 milliseconds, TR = 6.7 milliseconds, approximate TE = 3.0 milliseconds, flip angle = 11°, FOV = 256 × 256 mm^2^, matrix = 256 × 256, number of slices = 184, isotropic voxel size = 1.0 mm^3^) and a T2-weighted CUBE sequence (GE Healthcare; TR = 2500 milliseconds, TE = 60 milliseconds, echo train length = 100; FOV = 256 × 256 mm^2^, number of slices = 184, isotropic voxel size = 1.0 mm^3^). Neuromelanin-MRI images were visually inspected for artifacts immediately on acquisition with scans repeated when needed.

### Statistical Analysis

The preprocessing steps used to generate our final metric of CNR of the SN/VTA complex (hereby referred to as *neuromelanin-MRI signal*) are available in the eMethods in Supplement 1. All analyses were carried out in Matlab (MathWorks) using custom scripts. Linear mixed-effects analyses were performed across participants for every voxel *v* within the SN/VTA mask, as *CNR_v_* = β_0_ + β_1_ · *CUD* + β_2_ · *FES* + β_3_ · *age* + β_4_ · *sex* + β_5_ · *time* + *b*_0_*_s_* + ε. In region of interest (ROI) analyses, voxel-level CNR (*CNR_v_*) was replaced by CNR averaged across the whole ROI (CNR-ROI). Further analyses added an interaction term, CUD × FES, in the model. Symptom analyses included the appropriate severity measures (eg, PANSS negative scores) in the model.

In the voxelwise analyses, the spatial extent of an effect was defined as the number of voxels *k* (adjacent or nonadjacent) exhibiting a significant association between the measure of interest and CNR (voxel-level height threshold for *t* test of regression coefficient β_1_ of *P* < .05, 1-sided [β × 1]). Hypothesis testing was based on a permutation test in which the measure of interest was randomly shuffled with respect to CNR. This test corrected for multiple comparisons by determining whether an effect’s spatial extent *k* was greater than would be expected by chance (corrected *P* <.05; 10 000 permutations; equivalent to a cluster-level familywise error–corrected *P* value).

Two different SN/VTA subregions were examined for ROI analyses, psychosis voxels (defined in Cassidy et al^19^), and CUD voxels (defined as those showing a CUD effect above the voxel-level height threshold in voxelwise analysis in this dataset). As cannabis use can be linked more directly with psychotic experiences than with the diagnosis of schizophrenia per se,^31^ and given the complications of examining symptom severity (independently from treatment response) in our sample of treated patients, we focused on the previously defined mask of psychosis voxels for ROI analysis, seeking the association of CUD diagnosis and CUD × FES interaction with neuromelanin-MRI signal. Additionally, to assess the association between neuromelanin-MRI signal and increasing CUD severity, we conducted a regression analysis using CUD severity categorized as none, mild, or moderate/severe. Given the known association of cannabis use with negative symptoms and general psychopathology,^31^ we examined the association between these symptoms as well as positive symptoms and the mean neuromelanin-MRI signal, both in the CUD voxels and the psychosis voxels in supplemental analysis.

## Results

**Figure 1.**
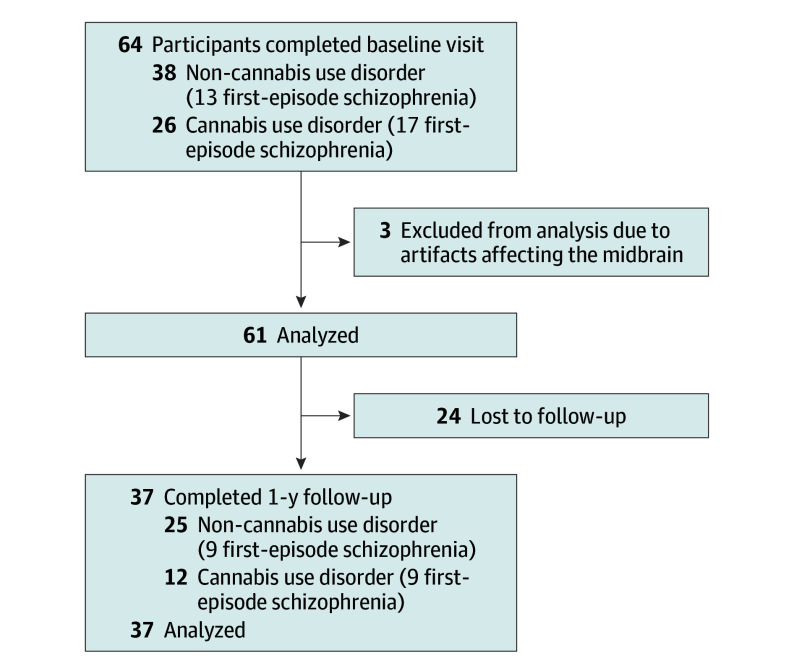
Study Flowchart

Baseline demographic and clinical characteristics are shown in the Table. A total of 36 individuals without CUD (mean [SD] age, 22.3 [3.2] years; 7 female [19%]; 29 male [81%]; 12 with FES) and 25 individuals with CUD (mean [SD] age, 24.3 [4.7] years; 3 female [12%]; 22 male [88%]; 16 with FES) participated in the study (Table and Figure 1). One-year follow-up was completed for 12 participants with CUD and 25 without CUD at a mean (SD) of 401.5 (49.7) days after baseline. There were no significant differences found between the group with CUD and the group without CUD regarding sex, age, or years of education (Table). The group with CUD had significantly lower premorbid IQ (mean [SD], 109.42 [5.57] vs 113.30 [5.15]; *P* = .007) and category fluency scores (mean [SD], 22.00 [5.86] vs 25.83 [5.86]; *P* = .01), but higher nicotine use (mean [SD] cigarettes per day, 3.30 [6.01] vs 0.51 [2.87]; *P* = .005) and salivary THC levels (mean [SD], 17.47 [33.70] vs 2.53 [10.20]; *P* = .01) compared with the group without CUD (Table). Those with CUD scored a mean (SD) 10.56 (3.22) of the possible 24 points on the cannabis-substance use questionnaire, with a mean (SD) onset age of 17.64 (2.93) years, suggesting that most individuals had a moderate dependence with less than 10 years’ duration of use (mean [SD] age, 24.32 [4.78] years) (Table). No significant differences were found in alcohol use frequency, age of regular cannabis use onset, or modified DSST scores (Table). There were more participants with FES in the CUD group than in the group without CUD, with trend-level significance (Table). Demographic/clinical characteristics for all groups can be found in the eTable in Supplement 1.

### Voxelwise Association Between Neuromelanin-MRI Signal, CUD, and Psychosis

**Figure 2.**
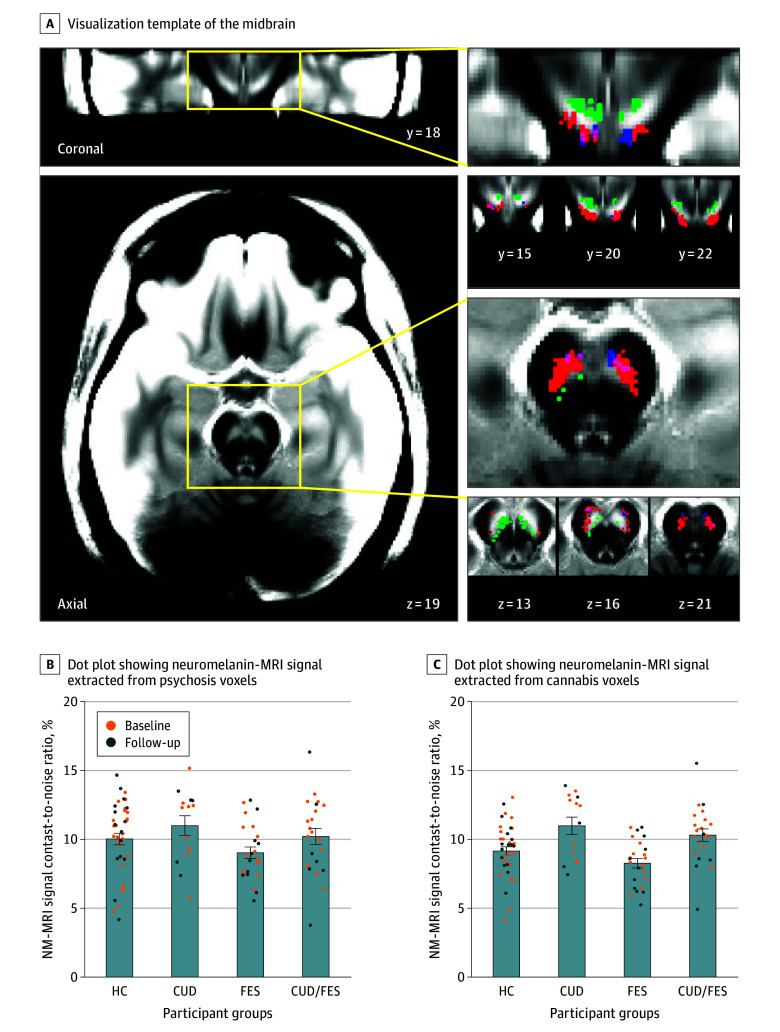
Substantia Nigra/Ventral Tegmental Area Neuromelanin-Sensitive Magnetic Resonance Imaging (NM-MRI) Signal in Participants With Cannabis Use Disorder (CUD) and First-Episode Schizophrenia (FES) A, Visualization template of the midbrain generated by averaging spatially normalized neuromelanin-MRI signal images from study participants. Magnifications show substantia nigra/ventral tegmental area voxels where the neuromelanin-MRI signal was elevated (red) in participants with cannabis use disorder (CUD) compared with individuals without CUD. Psychosis voxels previously shown to correlate with untreated positive symptoms of psychosis are shown in blue (overlap with cannabis-elevated voxels in violet). These voxels were clustered near the cannabis-elevated voxels. Green voxels are where the neuromelanin-MRI signal was reduced in participants with CUD compared with individuals without CUD. B, Dot plots showing the neuromelanin-MRI signal extracted from psychosis voxels in 4 groups, including healthy controls (HCs), those with a CUD, those with FES, and those with both a CUD and FES. Orange dots represent scans collected at baseline and blue dots at follow-up. C, Dot plots showing the neuromelanin-MRI signal extracted from cannabis voxels in 4 groups; orange dots were collected at baseline and blue dots at follow-up. Error bars represent standard error of the mean.

In a voxelwise analysis, neuromelanin-MRI signal was higher in participants with CUD compared with participants without CUD in a set of ventral SN/VTA voxels (387 of 2060 SN/VTA voxels, linear mixed-effects analysis controlling for FES diagnosis, age, sex, and time, corrected *P* =.03, permutation test; hereafter, the 387 voxels are referred to as *CUD voxels*) (Figure 2). There was also a set of voxels in the mediodorsal SN/VTA where neuromelanin-MRI signal was lower in participants with CUD, although this did not achieve significance (211 of 2060 SN/VTA voxels, corrected *P* =.15, permutation test) (Figure 2). On the other hand, in this model, FES diagnosis was not significantly associated with neuromelanin-MRI signal (241 of 2060 SN/VTA voxels showed increased signal in participants with FES, corrected *P* =.09, permutation test) (eFigure 1 in Supplement 1). Furthermore, there was no significant evidence of an interaction of CUD by FES (129 of 2060 SN/VTA voxels showed a positive interaction, corrected *P* =.30; 62 of 2060 SN/VTA voxels showed a negative interaction, corrected *P* =.47).

There was no association of time with neuromelanin-MRI signal in any group. The set of CUD voxels was of similar size when including nicotine use as a covariate (eResults in Supplement 1).

### ROI Analysis

Neuromelanin-MRI signal extracted from a mask of psychosis voxels was found to be significantly higher in participants with CUD compared with participants without CUD (*t*_92_ = 2.12, *P* = .04, linear mixed-effects model controlling for FES diagnosis, age, sex, and time). Further analyses controlling for nicotine use are available in the eResults in Supplement 1. There was no interaction between FES diagnosis and CUD in these voxels (*t*_55_ = 0.02), although the CUD association with the neuromelanin signal was numerically stronger (by 1.8 times) in those with FES (Cohen *d* = 0.71) compared with those without FES (Cohen *d* = 0.39) (Figure 2).

**Figure 3.**
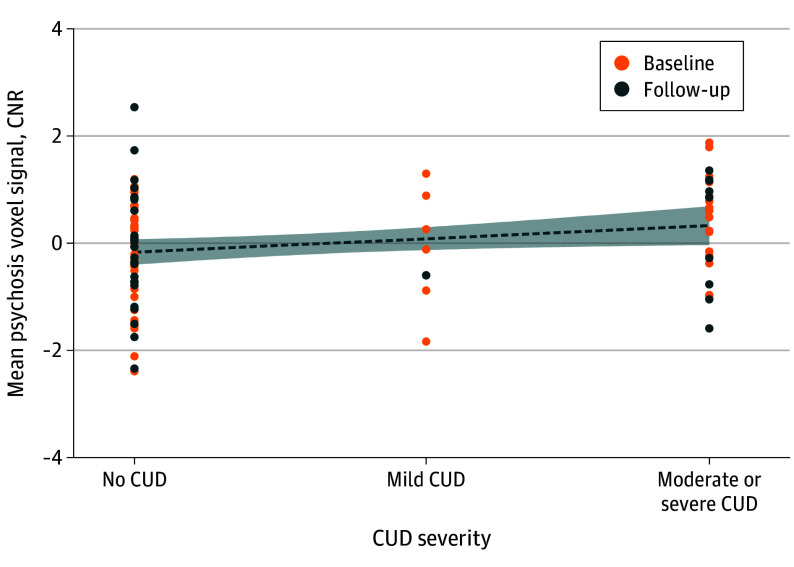
Psychosis Voxel Signal and Cannabis Use Disorder (CUD) Severity Correlation between CUD severity and mean neuromelanin-sensitive magnetic resonance imaging (neuromelanin-MRI) signal in psychosis voxels, corrected for age, sex, diagnosis, and time.

To test if increasing CUD severity linearly relates to a change in neuromelanin-MRI signal within the psychosis voxels, we conducted a regression analysis with CUD severity grouped as none, mild, or moderate/severe with baseline and follow-up data. This grouping, though arbitrary, provided the optimal distribution to test the dose effect. In the psychosis voxels, we found a significant dose-dependent association between neuromelanin-MRI signal and CUD (*F*_1, 96_ = 4.89; *P* = .03) (Figure 3). This indicates that an escalating severity of CUD may be associated with an increased neuromelanin signal in midbrain regions that are most sensitive to psychotic symptom burden. The results of CUD voxels neuromelanin-MRI signal and symptom scores are available in eFigure 2 in Supplement 1.

## Discussion

We investigated whether neuromelanin-MRI signal is altered in individuals with a CUD compared with individuals without a CUD, and whether CUD and FES exhibit an additive outcome with the neuromelanin-MRI signal. Consistent with our expectations, CUD was independently associated with higher neuromelanin-MRI signal in a ventral SN/VTA region. Additionally, individuals with CUD exhibited elevated neuromelanin-MRI signal in a subset of voxels previously associated with psychosis severity. Although we did not see a statistically significant additive outcome of schizophrenia and CUD with neuromelanin-MRI signal in our FES sample receiving early intervention, the association of CUD with neuromelanin-MRI signal was nearly 2 times higher in FES.

Given that neuromelanin-MRI signal in the ventral SN/VTA directly correlates with striatal dopamine synthesis^27^ and release capacity^19^ in schizophrenia, our findings of elevated signal in this region in CUD suggest that cannabis may be associated with the final common pathway of dopaminergic dysfunction relevant to psychotic symptoms. This is consistent with (1) clinical studies that link cannabis use with more pronounced positive symptoms in those with psychosis^9^ and (2) imaging studies in schizophrenia showing increased dopamine in the dorsal striatum, which receives SN/VTA dopaminergic projections.^15^ Thus, CUD-related dopamine turnover in the SN/VTA may influence psychosis-related striatal dopamine excess via the nigrostriatal pathway. Unlike prior studies of untreated psychosis,^32^ we did not see a higher neuromelanin-MRI signal in those with FES compared to healthy controls, but noted a numerical trend in the relevant voxels (eFigure 2 in Supplement 1).^27^ Alongside the lack of power, it is possible that the limited effect of FES on neuromelanin-MRI signal is due to treatment effect. Our sample of participants with FES had low symptom burden and were taking antipsychotics (eTable in Supplement 1 contains medication adherence and dose information), which influences dopamine turnover, possibly normalizing the higher neuromelanin-MRI signals.

Individuals with CUD have higher neuromelanin-MRI signal in psychosis voxels and in ventral SN/VTA voxels. We do not see a significant CUD-associated reduction in midbrain neuromelanin-MRI signal, in contrast to prior PET findings on striatal dopaminergic activity. Dopamine PET evidence stems from 1 case-control study^17^ assessing synthesis capacity, along with amphetamine challenge studies assessing release but with smaller sample sizes than our study (Xu and colleagues^33^ provide a detailed review). Participant profiles also vary between most PET studies and our sample. Our sample was younger, with an established dependence pattern and not excluded for psychotic disorders. Our participants with CUD scored a mean (SD) 10.56 (3.22) of the possible 24 on the cannabis substance use questionnaire, with a mean (SD) onset age of 17.64 (2.93) years, suggesting that most individuals had a moderate dependence with less than 10 years’ duration of use (mean [SD] age, 24.32 [4.78]). There is a need for both longitudinal data and multimodal (PET/neuromelanin-MRI) studies to develop a more complete causal mechanistic perspective of a CUD-psychosis link.^33^

In our sample, we did not see further changes in the SN/VTA neuromelanin-MRI signal with continued use of cannabis over 1 year. Neuromelanin-MRI signal within the SN/VTA reportedly increases with age.^34,35,36^ Our sample’s age range (17-35 years; mean [SD], 22.78 [3.96] years) was small and insufficient to capture age-related effects. This may also explain our null findings with relation to time effect. We also did not see any interaction among CUD, psychosis, and time, indicating that the baseline elevated neuromelanin-MRI signals persisted without normalizing by 1 year. Notably, individuals in the CUD group continued to satisfy persistent CUD at both time points in our sample, with no attrition of diagnosis. Consequently, any dopamine blunting related to persistent use might have already occurred before the initial evaluation (211 voxels with numerically, but not significantly, lower signal) (Figure 2), with no further decline over the 1-year window. Another plausible hypothesis is that a higher neuromelanin-MRI signal may predispose people to a CUD, but our study was not designed to address this question.

Neuromelanin, a product of dopamine auto-oxidation, is not completely inert.^37^ It binds to metals and toxic compounds that contribute to oxidation-reduction processes, and may prevent neuronal damage, partly as an antioxidant and scavenger of radical ions.^37^ Additionally, neuromelanin can function as an iron-binding molecule to regulate iron homeostasis in neuromelanin-containing neurons.^38^ Thus, we cannot infer if higher or lower levels of neuromelanin represent ill vs beneficial effects. We express caution in making such inferences especially in relation to cannabis and psychosis.

We chose to analyze a subset of voxels based on a previous neuromelanin study^19^ and examine the mean neuromelanin-MRI signal for specific SN/VTA subsets. We chose an external source to determine the psychosis voxel subset to prevent circular reasoning, ensuring the evidence for convergence with psychosis is independent of our dataset, and comes from observations in participants who have not taken medication. However, in our medicated sample with low symptom burden, the association with symptom severity has not been reproduced. Additionally, we used voxel subsets instead of a mean value for the entire SN/VTA, as distinct areas of the SN/VTA project to distinct regions of the nigrostriatal system^39^; this ensured that pathway-specific inferences were not obscured in our study.

### Strengths and Limitations

Our study has several strengths. We used means of voxel groups and voxelwise analyses of neuromelanin-MRI signal, ensuring a comprehensive evaluation of its distribution. We used multiple methods to examine cannabis use; CUDs were diagnosed by a psychiatrist, and we included both salivary THC and scores from the cannabis subscale of the substance use questionnaire, enhancing the reliability of the findings. Despite these strengths, certain limitations exist. Female participants are underrepresented in our sample. We restricted the study to SN/VTA, with no data for locus coeruleus, another neuromelanin-rich region. Our sample is relatively small and notably, the subsample with longitudinal data was powered only to detect large effect changes over time (effect size of 0.8 or greater); thus, the lack of 1-year change needs to be treated with caution. The generalizability of these findings may be limited by these factors and calls for further studies using our approach.

## Conclusions

In this cohort study, results suggest that people with CUD have a higher neuromelanin-MRI signal in brain areas associated with the expression of psychosis. This might explain how cannabis affects psychotic symptoms. The presence of numerically higher signal in certain voxels in FES despite their treatment status and numerically lower signal in certain voxels in CUD highlights the need for further examination of SN/VTA neuromelanin-MRI. Larger longitudinal studies can help us understand how cannabis use changes the dopamine system in psychosis over time.

## Notes

Supplement 1.
eMethods.

eResults.
**eTable.** Demographics and Clinical Characteristics of the Cannabis Use Disorder Group and Non–Cannabis Use Disorder Group, With and Without First-Episode Schizophrenia **eFigure 1.** Substantia Nigra/Ventral Tegmental Area Voxels in Which Neuromelanin-MRI Signal Was Numerically Elevated in First-Episode Schizophrenia Relative to Healthy Controls **eFigure 2.** Mean Neuromelanin-MRI Signal, Corrected With Age, Sex, Time, and Psychosis Diagnosis, in the Substantia Nigra/Ventral Tegmental Area Subregion Increased in Cannabis Use Disorder and the Relationship With PANSS Negative Scores
eReferences.

Supplement 2.
Data Sharing Statement.
