# Supplementary material for: Convergence of Cannabis and Psychosis on the Dopamine System
Source: JAMA Psychiatry. 2025 Apr 9;82(6):609–17. doi: 10.1001/jamapsychiatry.2025.0432 (PMC11983296; doi:10.1001/jamapsychiatry.2025.0432)
Supplement: Supplement 1. — eMethods. eResults. eTable. Demographics and Clinical Characteristics of the Cannabis Use Disorder Group and Non–Cannabis Use Disorder Group, With and Without First-Episode Schizophrenia eFigure 1. Substantia Nigra/Ventral Tegmental Area Voxels in Which Neuromelanin-MRI Signal Was Numerically Elevated in First-Episode Schizophrenia Relative to Healthy Controls eFigure 2. Mean Neuromelanin-MRI Signal, Corrected With Age, Sex, Time, and Psychosis Diagnosis, in the Substantia Nigra/Ventral Tegmental Area Subregion Increased in Cannabis Use Disorder and the Relationship With PANSS Negative Scores eReferences. [file jamapsychiatry-e250432-s001.pdf]

Supplementary Online Content

Ahrens J, Ford SD, Schaefer B, et al. Convergence of cannabis and psychosis on the dopamine system. *JAMA Psychiatry*. Published online April 9, 2025.
doi:10.1001/jamapsychiatry.2025.0432

eMethods.

eResults.

eTable. Demographics and Clinical Characteristics of the Cannabis Use Disorder Group and Non–Cannabis Use Disorder Group, With and Without First-Episode Schizophrenia

eFigure 1. Substantia Nigra/Ventral Tegmental Area Voxels in Which Neuromelanin-MRI Signal Was Numerically Elevated in First-Episode Schizophrenia Relative to Healthy Controls

eFigure 2. Mean Neuromelanin-MRI Signal, Corrected With Age, Sex, Time, and Psychosis Diagnosis, in the Substantia Nigra/Ventral Tegmental Area Subregion Increased in Cannabis Use Disorder and the Relationship With PANSS Negative Scores

eReferences.

This supplementary material has been provided by the authors to give readers additional information about their work.

© 2025 Ahrens J et al. JAMA Psychiatry.

eMethods

Participants

This study is part of a larger CIHR-funded project that aimed to recruit 90 participants to examine white matter microstructural integrity. The group without a cannabis use disorder (referred to as nCUD in this supplement) was recruited to represent the general population and resemble the cannabis use disorder (CUD) group in key sociodemographic variables except for the diagnosis of CUD. This means the nCUD group had several individuals who had prior exposure to cannabis and psychosis was not an exclusion criterion for both groups. To demonstrate a difference in neuromelanin-MRI signal between CUD and nCUD (anticipated recruitment ratio 0.7:1 for CUD:nCUD) of the same magnitude as demonstrated between highly symptomatic patients with schizophrenia and healthy controls (t_{31}=2.10, Cohen d = 0.754, as in Cassidy et al.'s work[1]), with 80% power and 5% type 1 error probability in a two-tailed test, we required 25 CUD and 35 nCUD patients. Our eventual sample was n=25 CUD and n=36 nCUD.

The diagnostic assessment of the SCID was repeated at 6 months and 12 months later to confirm the diagnosis of schizophrenia and CUD. Timeline Follow back method was used to collect detailed information about past and current use of cannabis in all subjects at baseline, 6 months, and 1 year. The two groups were stable over the 1 year, with all CUD subjects still satisfying the CUD criteria with no new diagnosis of CUD emerging in the nCUD group in this sample. CUD participants were grouped to have a mild, moderate, or severe CUD based upon the DSM-5 criteria for substance use disorders, specific to cannabis. If participants met two or three of the 11 DSM-5 symptoms, they were deemed to have a mild CUD, four or five as moderate, and six or more symptoms as severe[2]. Individuals with known progressive brain diseases (eg, demyelinating disorders), contraindications to MRI, significant head injury, seizures, or ongoing alcohol or stimulant drug use disorder were excluded.

Clinical and Cognitive Measures

Premorbid IQ was measured with the National Adult Reading Test (NART[3]). Alcohol use, cannabis use, nicotine use, and drug dependency were measured with the Alcohol Use Disorders Identification Test (AUDIT[4]), Cannabis Abuse Screening Test (CAST[5]), Fagerstrom Test for Nicotine Dependence (FTND[6]), and the Substance Use Questionnaire (SUQ[7]).

Neuromelanin-MRI Preprocessing

Neuromelanin-MRI scans were preprocessed using Matlab, ANTs, and SPM12 software to allow for voxelwise analyses in standardized Montreal Neurological Institute (MNI) space. Neuromelanin-MRI scans were coregistered to participants' T1-weighted scans and normalized to MNI space using ANTs[8]. The resampled voxel size of unsmoothed, normalized neuromelanin-MRI scans was 1 mm, isotropic. Intensity normalization and spatial smoothing with a 1 mm full-width-at-half-maximum Gaussian kernel were performed using custom MATLAB (MathWorks) scripts. Neuromelanin contrast-to-noise ratio (CNR) for each of the 2060 SN voxels (v) in each participant was calculated as change in neuromelanin-MRI signal intensity (I) to a reference region (RR), the crus cerebri, white-matter tracts known to have minimal neuromelanin: $CNR_v=(I_v - \text{mode}(I_{RR}))/\text{mode}(I_{RR})$.

A mask of the reference region and of the SN was adapted from our prior work[1]. The mode(I_{RR}) was calculated for each participant from a kernel-smoothing function fitted to a histogram of the distribution of all voxels in the mask. The resulting neuromelanin-MRI contrast-to-noise ratio maps were then spatially smoothed with a 1-mm full width at half maximum Gaussian kernel. For each participant, SN voxels with extreme values >99 or <1 percentile were censored.

Statistical Analyses

As mentioned in the main text, we (1) focused on the neuromelanin-MRI signal of the 'psychosis voxels' for a Region-of-Interest analysis and to study the dose effect of cannabis and (2) assessed if symptoms vary with the mean neuromelanin-MRI signal of the CUD voxels, where the most prominent increase in neuromelanin signal occurred in the voxelwise search in our sample. We also studied the relationship of symptoms with psychosis voxel signal but this mask, as defined in Cassidy et al.[1] (and replicated in Wengler et al.[9]), was derived from untreated

© 2025 Ahrens J et al. JAMA Psychiatry.

patients, whereas the patients in our sample were receiving antipsychotic treatments, we did not expect to see any symptom-related variations in this subregion.

eResults

eTable 1. Demographics and clinical characteristics of the cannabis use disorder group and non-cannabis use disorder group, with and without first episode schizophrenia.

	nCUD-FES (N=12)		CUD-FES (N=16)		nCUD-nFES (N=24)		CUD-nFES (N=9)		*P*-value
	**N**	**%**	**N**	**%**	**N**	**%**	**N**	**%**	
Male	10	83	14	88	19	79	8	89	0.89
Alcohol Frequency:									0.76
Never	1	8	4	25	3	12.5	0	0	
Monthly	5	41.7	7	44	9	37.5	4	44	
2-4 times per month	3	25	3	19	5	20.8	3	33	
2-3 times per week	2	16.7	1	6.3	5	20.8	2	22	
>4 times per week	0	0	0	0	2	8.3	0	0	
	**Mean**	**SD**	**Mean**	**SD**	**Mean**	**SD**	**Mean**	**SD**	
Age	23.0	2.17	24	5.1	22.0	3.61	24.33	3.74	0.43
Years of Education	13.33	1.73	12.8	1.96	15.40	2.65	16.81	2.59	0.0002
Premorbid IQ	108.85	3.67	107.38	6.29	115.52	5.72	113.05	3.87	0.0001
Nicotine use (cigarettes/day)	5.92	9.0	5.0	7.42	0	0	0	0	0.003
Category Fluency score[a]	21.33	8.14	16.8	5.26	25.83	5.86	22.0	5.86	0.0007
DSST score	45.42	9.76	44.47	8.80	65.15	12.20	61.18	10.86	<0.0001
GAF score	54.25	17.82	52.29	12.32					0.67
PANSS positive	10.66	3.58	13.73	4.45	7.00	0	7.00	0	<0.001
PANSS negative	11.42	3.29	13.2	5.16	7.00	0	7.00	0	<0.001
PANSS general	25.92	6.69	26.07	7.09	16.20	0.58	16.56	0.73	<0.001
SOFAS score	53.83	15.66	49.93	18.24					0.60
CGI-S score	4.36	1.63	4.29	1.03					0.92

© 2025 Ahrens J et al. JAMA Psychiatry.

CDSS total score	3.41	3.51	3.5	4.26					0.80
HAMD total score	1.48	2.60	1.44	1.94					0.97
Days since illness onset[b]	110.6	59.38	87.88	52.11					0.36
Rx adherence %	93.75	21.65	90.63	18.42					0.63
DDD	1.08	0.70	1.03	0.76					0.87
Salivary THC levels (ng/ml)[c]	5.92	9.0	16.82	32.09	0.32	1.38	8.5	9.8	0.11
Age of regular cannabis use onset[d]	17.86	2.67	17.12	3.15	17.69	3.71	18.56	2.79	0.66

Abbreviations: FES= first episode schizophrenia patients; HC= healthy controls; nCUD= without a cannabis use disorder; CUD= with a cannabis use disorder; N= number; SD= standard deviation; IQ= intelligence quotient; DSST= customised digit symbol substitution test (mean of oral and written scores); GAF= global functioning scale; PANSS= positive and negative syndrome scale; SOFAS= social and occupational functioning scale; CGI-S= clinical global impressions-severity; Rx= prescription; DDD= defined daily dose (of antipsychotic medications) calculated as per World Health Organisation's methodology; THC = tetrahydrocannabinol. CDSS: Calgary Depression Scale for Schizophrenia; HAMD: Hamilton Depression Scale-17 items.

[a]raw count; [b]equal to days between admission date to psychosis program and the date of the study visit; [c]PT-nCUD n=7, PT-CUD n=11, HC-nCUD n=19, HC-CUDn=4; [d]in years, PT-nCUD n=7, PT-CUD n=15, HC-nCUD n=13, HC-CUD n=10.

Interactions between time, CUD, and psychosis on neuromelanin-MRI signal

There was no effect of time on SN signal (46 of 2060 voxels decreased over time, corrected P =0.82, permutation test; follow-up N=37), nor a significant time by CUD [86 of 2060 voxels decreased, corrected P =0.52; 55 of 2060 increased, corrected P =0.76; nCUD n=25; CUD= 12], or time by FES interaction [138 voxels decreased over time, $p_{corrected}$=0.41; 27 voxels increased over time, corrected P =0.83; HC n= 19; FES n= 18], or interaction between CUD, psychosis diagnosis, and time [118 voxels decreased over time, corrected P =0.38, 48 voxels increased over time, corrected P =0.76]. Taken together, these results indicate that any effect of CUD and FES in neuromelanin-MRI signals are subregion-specific.

Association between CUD-related neuromelanin-MRI signal and symptom burden

The mixed model analysis revealed a trend between mean neuromelanin-MRI signal from 'CUD voxels' and PANSS negative scores, suggesting that with each point incremental change in PANSS negative score, the projected value of mean neuromelanin-MRI signal escalates by 0.136 units (under the condition that all other variables remain unchanged). This observed trend, though not achieving conventional significance (t_{93} = 1.79 P = 0.07; with age, sex, time, CUD status, and diagnosis as covariates), hints at a potential association worthy of further exploration (see eFigure 1). No such relationships were noted between neuromelanin-MRI signal of the 'CUD voxels' and PANSS positive (t_{93} = 0.48, P > 0.05) or general (t_{93} = 0.44, P > 0.05) symptoms.

No relationships were found between neuromelanin-MRI signal extracted from the 'psychosis voxels' and PANSS negative (t_{93} =0.98, P > 0.05), positive (t_{93}= -0.21, P > 0.05), or general (t_{93}= 0.33, P > 0.05) symptoms.

Taken together, the trend reported here calls for further investigations to clarify if the CUD-related increase in SN/VTA neuromelanin-MRI signal primarily contributes to a higher negative symptom burden, and whether this relationship is restricted to the midbrain regions that are most sensitive to cannabis use.

© 2025 Ahrens J et al. JAMA Psychiatry.

Controlling for nicotine use

When adding nicotine use status as an additional covariate and repeating the voxelwise linear mixed effects analysis to predict neuromelanin-MRI signal, there were 389 voxels showing elevated signal in CUD, corrected *P* =0.026. Adding nicotine use to the ROI analysis to predict neuromelanin-MRI signal in 'psychosis voxels' found CUD participants continued to have significantly elevated signal compared to nCUD (t_{91}=2.12, *P* =0.037, linear mixed effects model controlling for FES diagnosis, age, sex, nicotine use, and time).

Rationale for CUD Severity Groups

The grouping of CUD participants as none, mild, and moderate/severe for the analyses of neuromelanin-MRI signal and increasing CUD severity was chosen to balance sample size across groups while preserving meaningful distinctions in CUD severity. Unlike other substance use disorders, most people who satisfy cannabis use disorder criteria appear to fall in the 'Mild' category[10], necessitating this majority to be considered as a separate group. Furthermore, as shown in DSM-5 field surveys[11] (mild 2.85%, moderate 1.42%, and severe 2.0%) grouping moderate/severe makes a subgroup with comparable size to 'mild CUD' at the population level. Although the final distribution within the sample is not uniform (29, 6, and 17 participants), this method allowed for a regression analysis that captured the population distribution optimally, with sufficient sample weights for all the user groups. We used the term "optimal distribution" to refer to this compromise between maintaining statistical validity and ensuring that the groups are meaningfully representative of increasing CUD severity.

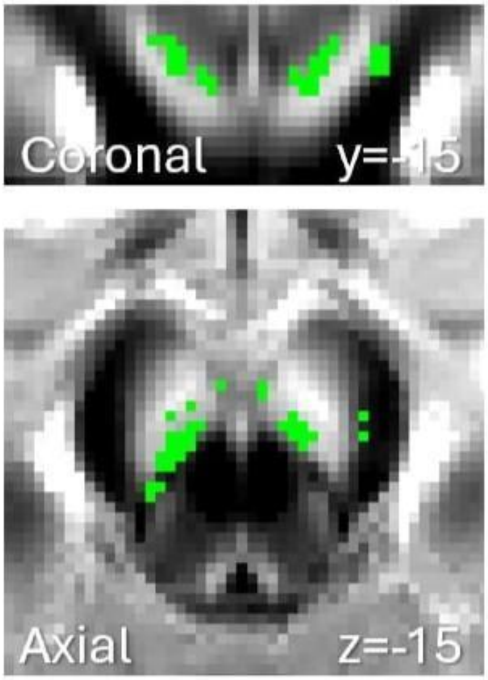

eFigure 1. Substantia Nigra / Ventral Tegmental Area voxels in which neuromelanin-MRI signal was numerically elevated in first episode schizophrenia relative to healthy controls (241 of 2060 SN/VTA voxels, increased signal in participants with FES, corrected *P* = 0.094, permutation test).

© 2025 Ahrens J et al. JAMA Psychiatry.

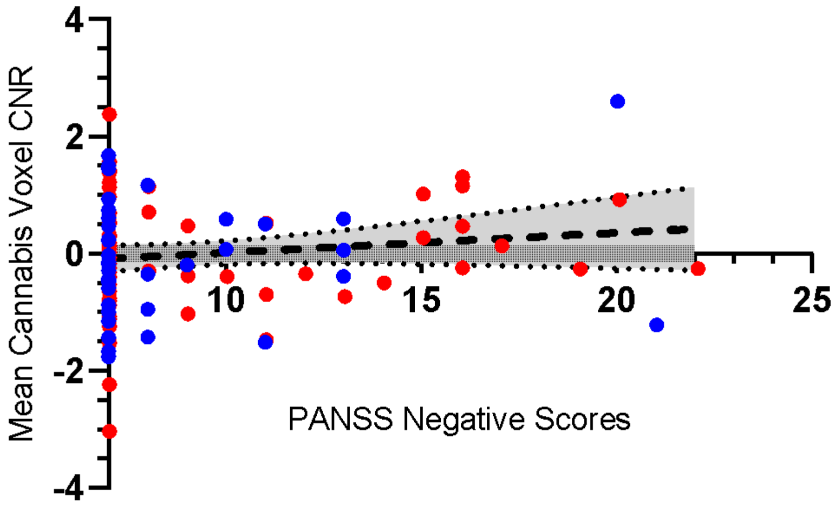

.

eFigure 2. Cannabis voxel signal and PANSS negative scores. Mean neuromelanin-MRI contrast-to-noise ratio, corrected with age, sex, and psychosis diagnosis, in the substantia nigra subregion increased in cannabis use disorder and the relationship with PANSS negative scores. Red dots are baseline values and blue dots are follow-up values. *Abbreviations:* CNR, contrast-to-noise ratio; PANSS, positive and negative syndrome scale.

eReferences

1. Cassidy CM, Zucca FA, Girgis RR, et al. Neuromelanin-sensitive MRI as a noninvasive proxy measure of dopamine function in the human brain. *Proc Natl Acad Sci U S A*. 2019;116(11):5108-5117. doi:10.1073/pnas.1807983116

2. American Psychiatric Association. *Diagnostic and Statistical Manual of Mental Disorders: DSM-IV*. Vol 4.; 1994.

3. Venegas J, Clark E. National Adult Reading Test. In: Kreutzer JS, DeLuca J, Caplan B, eds. *Encyclopedia of Clinical Neuropsychology*. Springer; 2011:1705-1705. doi:10.1007/978-0-387-79948-3_1467

4. Babor TF, Higgins-Biddle JC, Saunders JB, Monteiro MG. AUDIT: the alcohol use disorders identification test: guidelines for use in primary health care. *World Health Organization*. Published online 2001.

5. Legleye S, Piontek D, Kraus L. Psychometric properties of the Cannabis Abuse Screening Test (CAST) in a French sample of adolescents. *Drug and Alcohol Dependence*. 2011;113(2):229-235. doi:10.1016/j.drugalcdep.2010.08.011

6. Heatherton TF, Kozlowski LT, Frecker RC, Fagerstrom KO. The Fagerström Test for Nicotine Dependence: a revision of the Fagerstrom Tolerance Questionnaire. *British Journal of Addiction*. 1991;86(9):1119-1127. doi:10.1111/j.1360-0443.1991.tb01879.x

© 2025 Ahrens J et al. JAMA Psychiatry.

7. Molina BSG, Pelham WE. Childhood predictors of adolescent substance use in a longitudinal study of children with ADHD. *J Abnorm Psychol*. 2003;112(3):497-507. doi:10.1037/0021-843x.112.3.497

8. Wengler K, He X, Abi-Dargham A, Horga G. Reproducibility assessment of neuromelanin-sensitive magnetic resonance imaging protocols for region-of-interest and voxelwise analyses. *Neuroimage*. 2020;208:116457. doi:10.1016/j.neuroimage.2019.116457

9. Wengler K, Baker SC, Velikovskaya A, et al. Generalizability and Out-of-Sample Predictive Ability of Associations Between Neuromelanin-Sensitive Magnetic Resonance Imaging and Psychosis in Antipsychotic-Free Individuals. *JAMA Psychiatry*. 2024;81(2):198-208. doi:10.1001/jamapsychiatry.2023.4305

10. Budney, A.J., Sofis, M.J. and Borodovsky, J.T., 2019. An update on cannabis use disorder with comment on the impact of policy related to therapeutic and recreational cannabis use. European archives of psychiatry and clinical neuroscience, 269, pp.73-86.

11. Hasin DS, Kerridge BT, Saha TD, Huang B, Pickering R, Smith SM, Jung J, Zhang H, Grant BF (2016) Prevalence and correlates of DSM-5 cannabis use disorder, 2012–2013: findings from the national epidemiologic survey on alcohol and related conditions–III. Am J Psychiatry 173(6):588–599.

© 2025 Ahrens J et al. JAMA Psychiatry.
