# Supplementary material for: Convergence of Cannabis and Psychosis on the Dopamine System
Source: JAMA Psychiatry. 2025 Apr 9;82(6):609–17. doi: 10.1001/jamapsychiatry.2025.0432 (PMC11983296; doi:10.1001/jamapsychiatry.2025.0432)
Supplement: Supplement 2. — Data Sharing Statement. [file jamapsychiatry-e250432-s002.pdf]

Data Sharing Statement

Ahrens. Convergence of Cannabis and Psychosis on the Dopamine System. *JAMA Psychiatry*. Published April 09, 2025. doi:10.1001/jamapsychiatry.2025.0432

Data

Data available: Anonymized data presented in this study can be obtained upon reasonable request to the corresponding author, within the stipulations of the Research Ethics Board.
